# Types of Mankind

**Published:** 1854-09

**Authors:** 


Art. IV.—*Types of Mankind:* or Ethnological researches, based upon the Ancient Monuments, Paintings, Sculptures, and Crania of Races, and upon their Natural, Geographical, Philosophical, and Biblical history: illustrated by selections from the inedited papers of Samuel George Morton, M. D., (late President of the Academy of Natural Sciences at Philadelphia), and by additional contributions from Prof. L. Agassiz, LL. D; W. Usher, M. D.; and Prof. H. S. Patterson, M. D. By J. C. Nott, M. D., Mobile, Alabama, and George R. Gliddon, formerly U. S. Consul at Cairo. Philadelphia, 1854, Lippincott, Grambo, & Co.: pp. 738, 8vo.

Ethnology, or the Science of Races, is a branch of human knowledge which possesses interest for every class of readers. It touches every man, and involves questions of the greatest practical importance. Resting in part upon the sciences of Anatomy and Physiology, it challenges especially the attention of the physician; as a question of specific difference in the various races of men, it is one to excite the curiosity of the zoologist; and as it deals with the problem of man's origin upon the earth, it appeals with force to every inquisitive mind. It is not a question of science merely but one of religion also, and cannot be approached as a simple question in natural history, for it so happens that the "natural history" of man is indissolubly connected with his moral relations. No matter how much we may be disposed to divest the question of its psychological aspects, we attempt in vain to handle it as a question merely of time; its bearing upon the unseen future, upon the destiny of our race hereafter, will force itself upon the understanding. In regard to the animal races, when we have described the phenomena of their birth, their lives, and their dissolution, we have completed their history; but not so of the human race. When man reaches the goal of his earthly existence he resolutely demands, "*is this all?*" He trusts to enter then upon another state of being and to continue his existence forever.

It may be that the moral bearing of Ethnology has tended to deter inquirers from this field of investigation, and explains the backward state in which this science is still found. The study of the "Types of Mankind" involves, necessarily, the question of the unity of their origin. The scientific inquiry becomes, unavoidably, a theological question, in which most naturalists are little inclined to engage. But the investigation is going on; ethnology is making progress; the question of the unity of the human race is being continually started, and there is no course left but to meet it openly and candidly in fair discussion.

The work of which the title is given at the head of this article contains an elaborate exposition of the subject, and boldly assumes the heretical side of the question. Its numerous authors are agreed in this, that the various races of men have descended, not from a single pair, but from numerous parents. They reject, altogether, the account of man's creation given in the scriptures. They hold, not only, that the five varieties of Blumenbach constitute different species, but that several species are included in several of these families; in a word, that as the various zones of the earth have their appropriate races of animals, so the various tribes of men have been created on the continents or islands to which they pertain. They do not go quite so far as Dr. Robert Knox, a late writer on ethnology, who insists that men, like other animals, not only originate in particular localities, but, like them, are tied down, by a law of their nature, to the localities from which they sprung. They are not yet prepared to admit this, but they are far from pronouncing it, as an another advocate of their doctrine has done, "a groundless fallacy." They think the experiment of transplanting a tribe of men into a foreign soil, has not yet been fully tried; it is now going on, on a pretty large scale, in this country.

The differences between the different races of men, in form, features and complexion are so great as at once to attract the attention of the most careless observer. They cannot escape remark; every one is at once struck by them. The Indian, the Caucasian, and the African afford the most distinctive types, and in color and physiognomy these races differ so widely from each other, that one is not surprised at finding philosophers who insist that they form distinct species. How such diversity has been brought about, if not original; how peculiar types have been perpetuated since the earliest records of history; how the dispersion of the races has been accomplished in the period assigned by our common chronology, these are some of the great problems which the Ethnologist seeks to solve. The subject is environed with difficulties, and dogmatism is quite out of place in its discussion. We admit, with the ablest biblical critics, the difficulty of reconciling the proofs of the early existence of populous and highly civilized nations with the chronology founded upon the writings of Moses. We agree that it was not the purpose of the inspired historirn to give us an exact chronology, and that in his fragmentary record he has not even attempted a continuous genealogy. We are conscious of the injury done to the cause of sacred truth by a false interpretation of the scriptures, and are persuaded that "the unskilful insisting of our divines upon the literal sense of Moses," though it may not have "bred many hundred thousands of atheists," as some one has asserted, has been a fruitful source of infidelity. It will be wise in us of the present day in all controversies touching the divine record, if we avoid the error of those, in the time of Galileo, who staked the truth of the Bible against the Copernican system. The Copernican system has been universally received, and yet the faith of men in the truth of religion is as firm as ever.

BLUMENBACH, having with great labor collected the materials for a systematic account of the anatomical peculiarities of the different races of mankind, proposed a classification which has been generally adopted. He arranged them into five primary groups, chiefly according to color, and the configuration of the skull, and although it is admitted that these characters are not unvarying, none more permanent or distinctive have been discovered. The *Caucasian*, his first group, so called from Mount Caucasus, to which ancient traditions refer the origin of many celebrated nations, is made up of a great medley of races. Most of the European nations are included in this group, and the Georgian and Circassian tribes, the most beautiful of the human race in form and feature, are regarded as its true types. All writers on Ethnology belong to this fortunate branch of the human family. The second group is the *Mongolian*, a very numerous branch, embracing the inhabitants of China and Japan, and also the Magyar race of Hungary, of which Kossuth is a noted specimen The *Ethiopian* comprises the third group, taking in the tribes of Africa, and the fourth and fifth are the *Indian* and the *Malayan*.

Finding it impossible to assign to all these groups any definite types of cranial configuration, Dr. PRICHARD, the most learned and able of all ethnological writers, divides the human race into *three* leading types according to the shape of the cranium. In the first, which corresponds to the caucasian family of BLUMENBACH, the form of the skull is *oval* or *elliptical*, and of the most perfect symmetry. It is the head of the ancient Greeks, elevated, of great capacity, the face small in proportion, giving the impression of moral and intellectual elevation,—the head of the dominant races of men. The *pyramidal* skull is characteristic, not only of the Mongolian, but also of the Indian and Malayan tribes of BLUMENBACH, remarkable for their high cheek bones, the flatness of the upper half of the face, and foreheads tapering upwards, giving their heads from the cheek bones up a pyramidal form. The eyes are more deeply set, and the opening of the lids, in most instances, has a decided obliquity. The face is larger in proportion to the cranium than it is in the preceding race, indicating more space for the organs of sensation and less for the brain, which confers superiority upon the race. The nations constituting this group are for the most part nomadic, though they stretch from the icy sea of our continent to the southern parts of Africa, and embrace Indians and Esquimaux, as well as the Bushmen and Hottentots. Dr. PRICHARD's third type is the *prognathous*, marked by great forward prominence of jaws. It is found in great perfection in most of the Negro races, but is by no means confined to them, being almost as striking in some of the tribes of Australia and Polynesia. The head is compressed at the side, the forehead retreats, the jaws project forwards, and the occiput backwards. The undue prominence of the jaws gives them a reduced facial angle and an *animal* aspect, which produces at once in the beholder the idea of degradation.

If we turn from the form to the capacity of the cranium in these several groups of the human family, we shall find the difference to be very great. The Caucasian stands foremost, and exceeds all the rest as much in the size as in the symmetry of his head. Dr. S. G. MORTON measured 623 human crania, to ascertain the relative size of the brain in the various races, and the following are some of his results, as given in the third volume of the *Transactions of the American Medical Association.* The *Teutonic* race embracing Anglo-Saxons, Anglo-Americans and Anglo-Irish, possess the greatest cranial capacity; and the nations at the other extreme of the scale are the ancient Peruvians and the Australians. The present tribes of American Indians have larger heads than the demi-civilized Peruvians and Mexicans. The negro brain is nine cubic inches less than the Teutonic, but three larger than the ancient Egyptian. The largest cranium, that of a Dutch gentleman, in the collection of Dr. MORTON had a capacity of 114 cubic inches, but the head of Mr. WEBSTER measured 122 inches. The following is Dr. MORTON's table of admeasurements:—

| | Max. | Min. | Mean. | | | |
|---|---|---|---|---|---|---|
| German, | 114 | 70 | 90 | 18 | skulls | measured. |
| English, | 105 | 91 | 96 | 5 | " | " |
| American, | 97 | 82 | 90 | 7 | " | " |
| Mexican, | 92 | 67 | 79 | 22 | " | " |
| Peruvian, | 101 | 58 | 75 | 155 | " | " |
| Native African. | 99 | 65 | 83 | 62 | " | " |
| Hottentot, | 83 | 68 | 75 | 3 | " | " |
| Australian, | 83 | 63 | 75 | 8 | " | " |

As the sum of all the admeasurements made, we may take 92 cubic inches as the average cranial capacity of the Teutonic tribe, the highest branch of the Caucasian group; the Celtic, another branch of the same family, has a capacity of 87 cubic inches; the Negro and the Chinese have, each, 83; the barbarous tribes of American Indians have 84; and the Toltecan family, the Australian and Hottentot, only 75—a difference between the highest and lowest families of nearly 20 inches. The brains of the highest branch of the Caucasian race are larger by one fourth than the brains of the Malayan. And with the development of brain we mark an increasing capacity for mental culture and the refinements of civilization. All the nations of the earth that have become distinguished for intellectual advancement have belonged to the Caucasian race. The civilization, however, has not always been in strict proportion to the amount of brain; for it is evident from the memorials left behind them that the Toltecans, or Peruvians, once the dominant race of our continent, were a highly civilized people in comparison with the barbarous tribes of Indians by whom they were superseded. But the rule will generally hold, that the greater the cranial, the greater is the intellectual, capacity, and the greater the advancement in the social and moral scale. The nations of the *pyramidal* type of heads, as we have seen, are generally a barbarous, nomadic people; some of them wandering with their flocks and herds over the vast plains of Asia; others living by the chase, others supporting themselves by fishing along the shores of our northern seas, and others again subsisting upon insects, and such plants as grow spontaneously upon their native soil. And among the *prognathous* races, as the physiognomy leads us to expect, we find improvidence and destitution, an absence of all the arts that refine and elevate mankind, squalor, ignorance, and brutality.

But this brings us to the question whether these differences are so fixed and unvarying as to entitle them to be regarded as specific characters? A few details will be the most satisfactory answer to this question, and from them we shall be forced to infer, we think, that no specific distinction can be founded upon either the size or the shape of the cranium. As to shape, we believe we may safely appeal to common observation whether all the types of heads are not occasionally met with in the Caucasian family. Every day we meet with individuals, of undoubted Caucasian blood, exhibiting the pyramidal or prognathous form of head. And size is still less determinate. Who has not seen individuals of the African race with heads large and well-proportioned, and, on the other hand, members of the favored Caucasian race with brains so diminutive that they would scarcely fill the cranium of a Toltecan, Hottentot, or Malay? And as a nation, have we not seen that the ancient Egyptians, a branch of the Caucasian family, were not equal, according to Dr. Morton, in the size of their brains to the Negro race? Clearly then, as it appears to us, it is not philosophical to found a specific difference between families of the human race upon so varying a character as the form or size of the head.

Dr. Prichard, who has collected an immense body of testimony on this subject, remarks of the Mongolian group, that the peculiarities of the pyramidal skull are often found so much softened down, not in individuals alone, but in whole tribes of that race, as to approach the elliptical form; and states upon the authority of scientific travellers, that the same diversity prevails among the tribes which people Oceania. The skulls of the Malayan portion of the population are referable to the pyramidal type, but the savage races in and around Australia are as prognathous as the African Negroes; while "in many parts of the Polynesian Archipelago tribes of higher civilization are met with whose skulls can scarcely be distinguished from the best European forms." And so in regard to the indigenous races of our continent, if the pyramidal is the prevailing form of the skull, there are tribes which present a decidedly prognathous type, and others again that approach the elliptical form. M. D'Orbigny, a distinguished naturalist, speaking of what he has himself observed, says that "a Peruvian is more different from a Patagonian, and a Patagonian from a Guarini, than is a Greek from an Ethiopian or a Mongolian."

In regard to these facts one of two suppositions must be true; either that these tribes, inhabiting the same continent or group of islands, were originally of distinct stocks, or else that a transmutation may be effected, in the process of time, from one type to another. There are not wanting ethnologists who adopt the former explanation, and are ready to admit as many species as there are varieties of the human race; referring the aborigines of America as well as the Caucasian family, to "an infinite number of primitive stocks," and insisting that Celt, Saxon, Jew, Gipsey, Copt, Arabian, Norman, Hun, and Sarmatian, are specifically as well defined as Mongolian, Indian, Negro or Malayan. But let us inquire whether there are not facts which render the latter supposition highly probable, to say the least of it. In the work of Dr. Prichard to which we have referred, this laborious writer has collected abundant evidence of this change from one type to another. The Turks afford a striking example of this change of physiognomy, that branch of the nation in Europe having acquired so much of the Caucasian type as to have led many writers to place it in that group rather than in the Mongolian. And yet there is no room to doubt that both branches have descended from a common stock. It is in the western branch that the change has taken place—a change from a lower to a higher type—and the explanation of it is, that this branch has made decided advances in civilization and the arts, while the Asiatic branch has remained stationary.

A more remarkable instance is presented in the Magyar race, the nobility of Hungary, one of the finest races, physically and intellectually, in Europe. The evidence is clear to the mind of Dr. Prichard, who has investigated it carefully, that this superior race of men is a branch of the great Northern-Asiatic stock, and "is closely allied in blood to the stupid and feeble Ostiaks, and the untameable Laplanders." Expelled some thousand years ago, by the Turks, from Great Hungary, they in turn drove out the Slavonian nations from the fertile parts of Hungary, which they have continued to occupy. They exchanged a rigorous, inhospitable climate--the wilderness of the Samoiedes—for a country of genial climate and fertile soil, and changed their habits of life with the change of homes, adopting for their wild, savage pursuits, the peaceful employments of agriculture. And the result we behold in the elevation of the physical and moral man. In the ten centuries that have passed away since their migration, their heads have been gradually losing the pyramidal form and assuming the elliptical, until they have "become a handsome people, of fine stature and regular European features." The illustrious Hungarian exile, to whose eloquent addresses some of our readers have listened, is, as we have already remarked, a specimen of this race. Dr. Prichard thinks there is no reason for regarding this improvement as due in any considerable degree to an intermixture of races, for the Magyars have continued distinct from the other people of Hungary. We must ascribe it, we think, to the meliorating influences of civilization.

Are we not every day witnesses ourselves of a similar change in one of the races that make up our population? It is but a few centuries since the first Africans were brought to the shores of America, but we are sure we state the impression of many accurate observers when we say, that the descendants of the first Negroes have lost much of prognathous physiognomy of their ancestors. Our own observation agrees with that of the medical men who assured Sir Charles Lyell, during his recent tours in this country, that a gradual approximation is taking place, in the head and body of the Negroes, to the European model. We think we cannot be mistaken in the belief that each successive generation exhibits an improvement in these respects, especially in those of the race who live in close relation with their masters, and share to some extent in the advantages of education and an intellectual society. The contrast, not only in intelligence, but in form, gait, features, and expression, between such slaves, of certainly unmixed blood, and those who have been raised on plantations with but little intercourse with the whites, must have struck the most casual observer.

These are examples of change for the better—of an upward tendency of the race; unfortunately instances of the opposite tendency are far more numerous. "*Facilis descensus averni*," is a truth of which nations as well as individuals are constantly affording sad illustrations. The Irish who inhabit certain districts in Sligo, Mayo, and Leitrim, are the descendants of an athletic, comely race of men. Their fathers were driven by the British from Armagh and the south of Down, about two centuries ago. They have lived, generation after generation, in miserable hovels, dark, damp and unwholesome; subsisting upon a meagre diet, enslaved by ignorance and destitution. They are reduced in size to an average stature of little over five feet, and "are pot-bellied, bow-legged, and abortively featured; with open, projecting mouths, prominent teeth, and exposed gums; their advancing cheek-bones and depressed noses bearing barbarism on their very front." Such are the results, in one of the most exalted families of man, of the continued operation of such depressing causes as nakedness, hunger, ignorance and oppression. With these native Irish it has been brought about in the short space of two hundred years. There is no reason to doubt that under the same degrading influences the deterioration would continue to go on, until in time it would be difficult to distinguish individuals of this highest branch of the human race from the most degraded of the Mongolian or Malay. Indeed, the physical resemblance of these wretched Irish to the natives of Australia is already such as to strike every traveler. The effect of starvation and ill treatment is found to be the same upon every branch of the human family, tending to reduce the physical frame to the same low type and level.

From the foregoing facts, bearing upon the *specific* unity or diversity of the human races, the following conclusions, clearly stated by Dr. Carpenter, may be drawn:

"1. That the physical constitution of man is peculiarly disposed, like that of the domesticated animals, to undergo variations.

2. That the extreme variations which present themselves between the races apparently the most removed from one another, are not greater in *degree* than those which exist between the different kinds of domesticated animals, which are known to have descended from a common stock; and that they are of the same *kind* with the variations which present themselvss in any one race of mankind; the difference of *degree* being clearly attributable, in the majority of cases, to the respective conditions under which such race exists.

3. That none of the variations, which have been pointed out as existing between the different races of mankind, have the least claim to be regarded as valid specific distinctions; being entirely destitute of that fixity, which is requisite to entitle them to such a rank; and exhibiting, in certain groups of each race, a tendency to pass into the characters of some other.

4. That, in the absence of any valid specific distinctions, we are required, by the universally received principles of zoological science, to regard all the races of mankind as belonging to the same species."

If we turn from the physiognomy to the *color* of the different races of mankind, we shall find it equally wanting in the character of fixity. Can the Ethiopian change his hue, is a phrase generally believed to express a physical impossibility; and, so long as the Ethiopian continues to live under a burning sun, surrounded by all the physical and moral circumstances which pertain to his native home, we have no doubt his skin will remain unchanged. But is it irue, that each race of men does still preserve its original complexion, under all circumstances, in every change of climate? Let us look at some of the testimony on this subject.

The color of the skin was held by the older physiologists to depend upon a proper coloring tissue, termed the *rete mucosum*, which some anatomists fancied they had succeeded in demonstrating, and which was held to be much more amply developed in the dark-skinned than in the fair races; but later researches, aided by the microscope, have proved this to be an entire misconception of the structure. The color of the skin depends upon the epidermis, and the depth of the complexion is in proportion to the number and character of the pigment cells, the function of which is the generation of coloring matter of various hues. The shades of color vary with the amount and color of the pigment secreted.

Now, if we selected individuals of the several races presenting the typical complexion well-marked, it would be easy to make it appear that color affords sufficient grounds for a specific distinction. The white European, the black African, and the copper-colored Indian are separated so widely by this character that an instinctive belief is prone to refer them to a distinct parentage. We suppose that, in the absence of all prepossessions on the subject, the spontaneous deduction of the mind would be, that they were the offspring of different stocks. But when we examine more closely into the matter, and find the various races so shading off into each other that it becomes at last impossible to say where the white race ceases and the dark begins, we are compelled to give up this character also as one forming an impassable barrier between them. The distinction of color is not even maintained with constancy in the same race. We continually meet with individuals of the white race in whom the pigment secretion is so active, that they are not without some difficulty distinguished from the dark-skinned race; as, on the other hand, from the destitution of the pigment cells, we occasionally see *albinos* among Africans. This we observe in individual cases, but the remark is equally true in respect to tribes of men. For example, as stated by Dr. Prichard, in the great Indo-Atlantic family, which there is abundant reason to regard as having a common origin, we are presented with fair-skinned, blue-eyed races, and others of an olive hue, and others again who are decidedly black. Among the branches of the Syro-Arabian stock similar varieties obtain, those on the high lands of Arabia having light complexions, and, not unfrequently, blue eyes and red hair, whilst those near Muscat present a sickly yellow hue, and those of the lower countries bordering on the Nile are nearly jet black.

The Jews, a people of marked physiognomy, who have been often cited as an example of the permanence of physical characteristics, afford, on the contrary, a remarkable illustration of the influence of climate in diversifying the complexion. Where this race inhabits among Celts and Saxons, blue eyes and flaxen hair are not uncommon; in Portugal their complexion is very dark; and those who have descended from families long resident in Cochin and the interior of Malabar, are said to be "so black as not to be distinguishable by their complexion from the native inhabitants." Dr. Prichard mentions the interesting fact that a colony of Jews just arrived at a town in Cochin, are still so fair as to be called "White Jews," the climate not having had time to produce its natural effect upon the new immigrants.

If we turn from the fair races to the natives of Africa, we shall find that among these people the complexion is by no means the uniform black generally supposed; but, on the contrary, we have the best authority of travelers for saying, that the diversity of color among the different inhabitants of this vast continent is very great. Some of the Kafir tribes are represented as being of a light brown complexion and reddish hair, and having, with these, features approaching a European type. The Hottentot is rather of a yellow complexion than a jet black; the Fulahs of Central Africa are of a dark copper color, resembling the Indian; while the natives of Africa on the Red Sea bear as strong a resemblance to the ancient Egyptians as to the true Negro race.

Thus we see the same race of people presenting almost every shade of complexion, the white and the black types so melting into each other that it becomes difficult to draw the line of separation between them. The third specific test has been proposed—the character of the *hair*—is a much less reliable test than either of the others, and therefore will detain us but a short time.

Until the microscope revealed its true structure, the hair was believed to afford a valid criterion by which races might be separated from each other. The covering on the Negroe's head was maintained to be *wool* and not hair; but microscopic investigations have put this distinction to flight. The intimate structure of the Negroe's hair differs in nothing from that of the white and Indian races, save in the greater quantity of pigment which it contains. Nor is the hair of the darkest of these people always crisp and twisted, any more than that of the white race is always straight and flowing. We often meet with individuals belonging to the Caucasian family with hair so curly and frizzled as closely to resemble that of the African; and on the other hand we now and then encounter jet black Africans with smooth, flowing hair.

Other parts of this argument we shall be compelled to pass over with a single remark or two.

The physiological and psychological evidence in favor of the unity of the human race is such as few minds that have weighed it impartially have been able to gainsay or resist. An average duration of life among all the races nearly the same; the correspondence between the various epochs of life; the same duration of pregnancy; the same intervals of the catamenia, and of the time of their irruption and final cessation; the subjection of all the families of man to the same diseases, whether sporadic, contagious, or epidemic; and, finally, the fruitfulness attending the intercourse of all the races with each other—these are physiological facts which can be affirmed of no truly distinct species among the lower animals. Do they not proclaim the family of man to be one?

The psychological argument is so happily summed up by Dr. Prichard, in his Natural history of Man, that we adopt his words: "We contemplate, among all the diversified tribes, who are endowed with reason and speech, the same internal feelings, appetencies, and aversions; the same inward convictions, the same sentiments of subjection to invisible powers, and (more or less fully developed) of accountableness or responsibility to unseen avengers of wrong, and agents of retributive justice, from whose tribunal men cannot even by death escape. We find everywhere the same susceptibility, though not always in the same degree of forwardness or ripeness of improvement, of admitting the cultivation of those universal endowments, of opening the eyes of the mind to the more clear and luminous views which Christianity unfolds, of becoming moulded to the institutions of religion and of civilized life; in a word, the same inward and mental nature is to be recognized in all the races of men."

The doctrine of the *diverse* origin of the races is urged in the work before us at great length, and with equal earnestness, and is sustained by a mass of historical testimony such as was never before presented on the subject. We have no hesitation in saying that it is much the ablest work yet produced on that side of the question. It is the production, as its title shows, of numerous pens. After a memoir of the lamented Morton, by Dr. Patterson, the first paper is a "Sketch of the Natural Provinces of the Animal World and their relation to the different Types of Man," by Prof. Agassiz. The aim of this paper is to show, that the range of the distinct types of the human race and of certain combinations of animals is the same; in other words, that each type of the race is associated with its peculiar *fauna*, and the theory is happily illustrated by a diagram at the end of the paper. Under the head *Negro*, we have, in this table, monkey, elephant, rhinoceros, hippopotamus, wart-hog, and giraffe, constituting the *types* of the fauna of Africa. Associated with the *Australian* race, we find the marsupial animals, the ant-eater, and the ornithorhynchus, as the proper fauna of that region; and so on. It may be that the learned author of this ingenious "Sketch" sometimes pushes a principle too far, but no one can read his generalizations on this subject without being struck with the highly philosophical character of this paper. As, to our minds, the most interesting chapter in this large book, we shall give the principal deductions of Prof. Agassiz on this subject. After referring to the fauna of the poles, he says:

"To the glacial zone, which incloses a single fauna, succeeds the temperate zone, included between the isothermes of 32°, and 74° Fahr., characterized by its pine forests, its amentacia, its maples, its walnuts, and its fruit trees, and from the midst of which arise like islands, lofty mountain chains or high table-lands, clothed with a vegetation which, in many respects, recalls that of the glacial regions. The geographical distribution of animals in this zone, forms several closely connected, but distinct combinations. It is the country of the terrestrial bear, of the wolf, the fox, the weasel, the marten, the otter, the lynx, the horse and the ass, the boar, and a great number of stags, deer, elk, goats, sheep, bulls, hares, squirrels, rats, &c.; to which are added southward, a few representatives of the tropical zone.

"Wherever this zone is not modified by extensive and high table-lands and mountain chains, we may distinguish in it four *secondary* zones, approximating gradually to the character of the tropics, and presenting therefore a greater diversity in the types of its southern representation than we find among those of its northern boundaries. We have first, adjoining the arctics, a *sub-arctic* zone, with an almost uniform appearance in the old as well as the new world, in which pine forests prevail, the home of the moose; next, a *cold temperate zone*, in which amentaceous trees are combined with pines, the home of the fur animals; next, a *warm temperate zone*, in which the pines recede, whilst to the prevailing amentaceous trees a variety of evergreens are added, the chief seat of the culture of our fruit trees, and of the wheat; and a *sub-tropical zone*, in which a number of tropical forms are combined with those characteristic of the warm temperate zone. Yet there is throughout the whole of the temperate zone one feature prevailing; the repetition, under corresponding latitudes, but under different longitudes, of the same genera and families, represented in each botanical or zoological province by distinct so-called *analogous or representative species*, with a very few subordinate types, peculiar to each province; for it is not until we reach the tropical zone that we find distinct types prevailing in each fauna and flora. Again, owing to the inequalities of the surface, the secondary zones are more or less blended into one another, as, for instance, in the table-lands of Central Asia, and Western North America, where the whole temperate zone preserves the features of a cold temperate region; or the colder zones may appear like islands rising in the midst of the warmer ones, as the Pyrenees, the Alps, &c., the summits of which partake of the peculiarities of the arctic and sub-arctic zones, whilst the valleys at their base are characterized by the flora and fauna of the cold or warm temperate zones."

The temperate zone he divides into an *Asiatic*, a *European*, and an *American* realm, the fauna of which is migratory as the climate is variable.

"The temperate zone is not characterized, like the arctic, by one and the same fauna; it does not form, as the arctic does, one continuous zoological zone around the globe. Not only do the animals change from one hemisphere to another, but these differences exist even between various regions of the same hemisphere. The species belonging to the western countries of the old world are not identical with those of the eastern countries. It is true that they often resemble each other so closely, that until very recently they have been confounded. It has been reserved, however, for modern zoology and botany to detect these nice distinctions. For instance, the coniferæ of the old world, even within the sub-arctic zone, are not identical with those of America. Instead of the Norway and the black pine, we have here the balsam and the white spruce; instead of the common fir, the *Pinus rigida;* instead of the European larch, the hacmatac, &c.; and farther south the differences are still more striking. In the temperate zone proper, the oaks, the beeches, the birches, the hornbeams, the hophornbeams, the chestnuts, the buttonwoods, the elms, the linden, the maples, and the walnuts, are represented in each continent by peculiar species differing more or less. Peculiar forms make here and there their appearance, such as the gum-trees, the tulip-trees, the magnolias. The evergreens are still more diversified,—we need only mention the camelias of Japan, and the kalmias of America as examples. Among the tropical forms extending into the warm temperate zone, we notice particularly the palmetto in the Southern United States, and the dwarf chamærops of southern Europe. The animal kingdom presents the same features. In Europe we have, for instance, the brown bear; in North America, the black bear; in Asia, the bear of Tubet: the European stag, and the European deer, are represented in North America by the Canadian stag, or wapiti, and the American deer; and in eastern Asia, by the musk deer. Instead of the mouflon, North America has the big-horn or mountain sheep, and Asia the argali. The North American buffalo is represented in Europe by the wild auerochs of Lithuania, and in Magnolia by the yak; the wild-cats, the martens and weasels, the wolves and foxes, the squirrels and mice (excepting the imported house mouse), the birds, the reptiles, the fishes, the insects, the mollusks, &c., though more or less closely allied, are equally distinct specifically. The types peculiar to the Old or New world are few; among them may be mentioned the horse and ass, and the dromedary of Asia, and the opossum of North America; but upon this subject more details may be found in every text book of zoology and botany. We would only add, that in the present state of our knowledge we recognize the following combination of animals within the limits of the temperate zone, which may be considered as so many distinct zoological provinces or faunæ.

"In the *Asiatic realm*,—1st, a northeastern fauna, the *Japanese fauna;* 2d, a southeastern fauna, the *Chinese fauna* and a central fauna, the *Magnolian fauna*, followed westwards by the *Caspian fauna*, which partakes partly of the Asiatic and partly of the European zoological character; its most remarkable animal, antelope saiga, ranging west as far as southern Russia. The Japanese and the Chinese faunæ, stand to each other in the same relation as southern Europe and north Africa, and it remains to be ascertained by farther investigations, whether the Japanese fauna ought to be sud-divided into a more eastern insular fauna, the *Japanese fauna proper*, and a more western continental fauna, which might be called the *Mandshurian* or *Tongousian* fauna."

The nations of men inhabiting those regions all belong to the Mongolian race, the natural limits of which correspond to the range of the Japanese, Chinese, Mongolian, and Caspian faunæ taken together.

The animals of the temperate zone of Europe Prof. A. divides into eight faunæ—a Scandinavian, a Russian, a Central European, one of southern Europe, one of Iran, a Syrian, an Egyptian, and the fauna of Atlas. "And here again," he remarks, "it cannot escape the attention of the careful observer that the European zoological realm is circumscribed within exactly the same limits as the so-called white race of man, including, as it does, the inhabitants of southwestern Asia, and of north Africa, with the lower parts of the valley of the Nile." It is curious :o remark farther, as he states, that the different sub-divisions of this race cover precisely the same ground as the special zoological provinces.

Of the fauna of our temperate zone he remarks—

"Though temperate America resembles closely, in its animal creation, the countries of Europe and Asia belonging to the same zone, we meet with physical and organic features in this continent which differ entirely from those of the Old World. The tropical realms, connected with those of the temperate zone, though bound together by some analogies, differ essentially from one another. Tropical Africa has hardly any species in common with Europe, though we may remember that the lion once extended to Greece, and that the jackal is to this day found upon some islands in the Adriatic, and in Morea. Tropical Asia differs equally from its temperate regions, and Australia forms a world by itself. Not so in southern America. The range of mountains which extends, in almost unbroken continuity, from the Arctic to Cape Horn, establishes a similarity between North and South America, which may be traced also, to a great degree, in its plants and animals. Entire families which are peculiar to this continent have their representatives in North, as well as South America, the cactus and didelphis, for instance; some species, as the puma, or American lion, may even be traced from Canada to Patagonia. In connection with these facts, we find that tropical America, though it has its peculiar types, as characteristic as those of tropical Africa, Asia, and Australia, does not furnish analogues of the giants of

Africa and Asia; its largest pachyderms being tapirs and pecaries, not elephants, rhinoceroses, and hippopotami; and its ruminants, the llamas and alpacas, and not camels and giraffes; whilst it reminds us, in many respects, of Australia, with which it has the type of marsupials, in common, though ruminants and pachyderms, and even monkeys, are entirely wanting there. Thus, with due qualification, it may be said, that the whole continent of America, when compared with the corresponding twin-continents of Europe-Africa or Asia-Australia, is characterized by a much greater uniformity of its natural productions, combined with a special localization of many of its subordinate types, which will justify the establishment of many special faunæ within its boundaries.

"With these facts before us, we may expect that there should be no great diversity among the tribes of man inhabiting this continent; and, indeed, the most extensive investigation of their peculiarities has led Dr. Morton to consider them as constituting but a single race, from the confines of the Esquimaux down to the southernmost extremity of the continent. But, at the same time, it should be remembered that, in accordance with the zoological character of the whole realm, this race is divided into an infinite number of small tribes, presenting more or less difference one from another.

"As to the special faunæ of the American continent, we may distinguish, within the temperate zone, a *Canadian fauna*, extending from Newfoundland across the great lakes to the base of the Rocky Mountains, a fauna of the *North American table-land*, a fauna of the *Northwest coast*, a fauna of the *middle United States*, a fauna of the *southern United States*, and a *California fauna*."

It is remarkable, when we consider how perfectly America is isolated from the continents of the Old World, that the correspondence should be so exact between the animals of our north temperate zone and those of Europe—the bear, the stag, the antelope, the goat, the sheep, and a species of the *bos* family entering into the fauna of each. There are only a few subordinate genera, of which the skunk and the opossum are examples, that are peculiar to this region of the American continent.

In tropical America the case is entirely different, there being there several distinct faunæ, many individuals of which have no representatives in the Old World. Its edentata and monkeys are peculiar; among pachyderms it has pecaries, which are not found elsewhere, and its marsupials differ entirely from those of Australia, as its ostriches do from those of all other countries.

"If we compare further the continents of the Old World with one another, we find a certain uniformity between the animals of Africa and tropical Asia. They have both elephants and rhinoceroses, though each has its peculiar species of these genera, which occur neither in America nor in Australia; whilst ceropitheci and antilopes prevail in Africa, and long-armed monkeys and stags in tropical Asia. Moreover, the black orangs are peculiar to Africa, and the red orangs to Asia. As to Australia it has neither monkeys nor pachyderms, nor edentata, but only marsupials and monotremes. We need therefore not carry these comparisons further to be satisfied that Africa, tropical Asia, and Australia constitute independent zoological realms.

"The continent of Africa south of the Atlas has a very uniform zoological character. This realm may however be subdivided, according to its local peculiarities, into a number of distinct faunæ. In its more northern parts we distinguish the fauna of the Sahara, and those of Nubia and Abyssinia; the latter of which extends over the Red Sea into the tropical parts of Arabia. These faunæ have been particularly studied by Ruppell and Ehrenberg, in whose works more may be found respecting the zoology of these regions. They are inhabited by two distinct races of men, the Nubians and Abyssinians, receding greatly in their features from the woolly-haired Negroes with flat broad noses, which cover the more central parts of the continent. But even here we may distinguish the fauna of Senegal from that of Guinea and that of the African Table land. In the first, we notice particularly the chimpanzee; in the second, the gorilla. There is no anthropoid monkey in the third. The fifth column of our Tableau gives figures of the most prominent animals of the genuine West African type. A fuller illustration of this subject might show, how peculiar tribes of Negroes cover the limits of the different faunæ of tropical Africa, and establish in this respect a parallelism between the nations of this continent and those of Europe."

The East Indian realm is the region of monkeys, which are not only its characteristic animals, but are here more diversified than they are found in any other quarter of the globe. Upon Borneo alone, an island not larger than Spain, eleven species exist, including the orang-outau.

Every reader has some knowledge of the wonders of the zoology of New Holland, its animals differing so completely from those of all other parts of the globe, as to constitute it a world in itself. Its extremely characteristic animals all belong to two families of the mammalia, the most humble of the class—the marsupials and monotremes. Its birds are strange, and were once gigantic. It has neither edentata nor ruminants, pachyderms nor monkeys, but yet it has its "two races of men, the Australian in New Holland, and the Papuan upon the Islands."

No one, we think, after a review of these remarkable facts, could resist the conclusion at which Prof. AGASSIZ arrives, "that the presence of animals upon the earth is not determined by physical conditions, but established by the direct agency of a Creator." And if the arrangement of coal and iron ore and the position of strata in the earth are held to afford proofs of benevolent design, we see not why this distribution of animal species according to their fitness for particular places of abode should be esteemed a less striking evidence of Divine beneficence and wisdom. But we are not prepared to admit the other deduction, inferable from the premises of this distinguished naturalist, rather than distinctly announced by him as his belief, that "the races of men were primitively created in nations," and in the zones of which, for the most part, they are still the inhabitants. We see no end to the number of primitive, independent stocks that must be admitted if we once yield the doctrine of the unity of the race. We have *two* distinct stocks, as we have seen, for Australia, a country that affords hardly a dozen species of the mammalian family. The "European zoological realm" of Prof AGASSIZ embraces no less than *four* races, the Semitic, the Græco-Roman, the Celto-Germanic, and the Sclavic, all of which, we are to suppose, had "an independent origin." Each fauna of Africa is described as having at its head a specific tribe of Negroes; and within the boundaries of the East Indian realm, we are assured, there are "*three* distinct races." With the progress of science the multiplication of species is likely to go on, until, while the horse, the camel, the ox, and the sheep, lay a doubtful claim to more than one original each, the number of distinct races among men must become almost infinite.

Dr. ROBERT KNOX, of London, has already advanced to this point, and is confident that America will ultimately be depopulated because the nations now in possession of it are not its native inhabitants. Each race, he holds, is tied down eternally to its own proper zone.

"Under the influence of climate," he says, "the Saxon decays in Northern America, and in Australia he rears his offspring with difficulty. He has changed his continental locality, a physiological law is against his naturalization there. Were the supplies from Europe not incessant, he could not stand his ground in these new continents. A *real native* permanent American or Australian race of pure Saxon blood, is a dream which can never be realized."

Look at the people of the United States, especially the women, he continues, and behold the depreciation!

"Already the United States man differs in appearance from the European; the ladies early lose their teeth; in both sexes the adipose cellular cushion interposed between the skin and the aponeuroses and muscles disappears, or at least loses its adipose portion; the muscles become stringy, and show themselves; the tendons appear on the surface; symptoms of decay manifest themselves. Now, what do these signs, added to the uncertainty of infant life in the Southern States, and the smallness of their families in the Northern, indicate? Not the conversion of the Anglo-Saxon into the red Indian, but warnings that the climate was not made for him, nor he for the climate."

It is true that all ethnologists who hold to the original diversity of the human race do not go so far as Dr. KNOX. A late American writer on the subject, Dr. CALDWELL, who in the main subscribed to all the sentiments of Dr. KNOX, denounced this conclusion, to which they seem naturally to lead, in the strongest terms. It is a "calumny" he said, "worn out by time," "overthrown and trampled on, upwards of sixty years ago, at the school where I received the rudiments of my English education." He declared the doctrine to be "stale, obsolete, and *repudiated*, if not actually forgotten, as unworthy of remembrance, by all enlightened and liberalized minds." Dr. KNOX, he added, in drawing "this disgusting and offensive picture" of the men and women of America, was guilty of "unsurpassed and unsurpassable mendacity;" and he expressed a doubt whether his brother ethnologist, while writing his book "was not either *monomaniacal* or *drunk*!"

Surely if ethnologists speak in this wise of the doctrines of one another, mankind in general may be excused for hesitating before they embrace them. We agree with Dr. CALDWELL, that a man has approached very near to the verge of insanity when he has persuaded himself that the Anglo-Saxon race is tending to extinction in America. But the learned authors of "Types of Mankind"

are by no means sure that Dr. KNOX is not right in his assertion. "Nature," they correctly say, "works not through a few generations, but through thousands of years," and "it is impossible to conjecture what time may effect." They add, "it would be a curious inquiry to investigate the physiological causes which have led to the destruction of ancient empires, and the disappearance of populations, like Egypt, Assyria, Greece and Rome. Many ancient nations were colonies from distant climes, and may have wasted away under the operation of laws that have operated slowly but surely." These authors are evidently not without misgivings that our great nation is destined to waste away, eventually, under the operation of this "physiological law" of the ethnologists.

Prof. AGASSIZ closes his lucid exposition, admirable equally for its broad scientific views and its calm, philosophical tone, with the remark, that in view of this question there are but two alternatives before us, viz:

"1st. Either mankind originated from a common stock, and all the different races with their peculiarities, in their present distribution, are to be ascribed to subsequent changes—an assumption for which there is no evidence whatever, and which leads at once to the admission that the diversity among animals is not an original one, nor their distribution determined by a general plan, established in the beginning of the Creation;—or

"2nd. We must acknowledge that the diversity among animals is a fact determined by the will of the Creator, and their geographical distribution part of the general plan which unites all organized beings into one great organic conception; whence it follows that what are called human races, down to their specialization as nations, are distinct primordial forms of the type of man."

Besides being opposed, as he alleges, "to all the modern results of science," Prof. AGASSIZ objects to the first of these alternatives, on the ground that "the consequences run inevitably into the Lamarkian development theory." Now it is curious that while this would seem to be the natural tendency of this doctrine, the very opposite is found to be the fact, the believers in the "development theory" being, according to our observation, those who also hold to the original diversity of the races of mankind. We have more than once mentioned the name of Dr. KNOX. He is represented as being a learned, and is unquestionably a bold, ethnologist; and we find him avowing his assent to this theory in language as plain as he can use. He adopts the words of GEOFFROY ST. HILAIRE, that "there is but *one* animal, not many," that "*nothing was created as it is; all is development from a microscopic point.*" And this is well known to be equally the doctrine of SPIX, OKEN, and VON MARTIUS, names of the very highest authority with the ethnologists of this school. When we hear this writer further asserting, that "of man's origin we know nothing correctly," and find him questioning the benefits of Christianity, can it be a matter of surprise that the theory which conducts him to such conclusions should excite opposition in serious minds—nay, that it should be almost universally rejected? Principles are fairly estimated by the results to which they lead. Tried by this test, we must say that there is nothing in the *philosophical* theory of man's origin to recommend it to our faith. We do not denounce the theory; by the strength of evidence alone it must stand or fall. If it be the true doctrine it will ultimately triumph; but as it now stands we are compelled to say, that the absurdities in which its advocates have hitherto involved it furnish to our minds strong reasons for regarding it with grave distrust.

We have remarked upon the excellent tone which pervades the contribution of Prof. AGASSIZ to this volume. It is such as becomes a discussion involving questions so momentous, and in which the evidence is so far from being all on one side that the great majority of scientific men are arrayed against him. The same cannot be said of the temper in which some of the other contributors to the work have conducted the controversy. Some of the writers exhibit what we cannot help regarding as an overweening confidence in the truth of their positions, and indulge in a large amount of dogmatic assertion. The vexed question of the unity of the race they do not consider as any longer *sub judice;* they seem out of patience with the dullness of those who hesitate to embrace their theory; and brush aside the cherished doctrine of their conscientious opponents—the "*universally received doctrine*" according to Prof. AGASSIZ—as unceremoniously as if it were some mere scientific speculation. We submit, respectfully, that it is not in the best taste to denounce as "a vulgar error" a belief held as sacred by the vast majority of serious men, and deemed defensible by most men of science. There may possibly be disputes in which offensive epithets are allowable, but surely a scientific inquiry like that before us is not one of the sort; and we cannot help thinking that such phrases as "sapient advocate," "mendacious instances," "illiterate advocates," &c., are unworthy of so grave a discussion. These writers are thoroughly persuaded of the truth of their position; the "*unity*-doctrine," they assert, is "shattered by Egyptian archæology, from the weather-vane to its foundations." It may be so, but undeniably the great body of scholars, and of men who have made physiology and zoology the study of their lives, are by no means convinced of the fact; and until the doctrine is more fully established, it strikes us that a tone a little less arrogant would be more becoming.

It would be easy to cite many examples of confident assertion upon points where the testimony is far from being conclusive, but we will content ourselves with a reference to a few which we have marked in the chapter, by Dr. Usher, on "*Geology and Paleontology in connection with human origins.*" Referring to some human bones found on the coast of Brazil, Dr. Usher exclaims:—

"Here, then, is one '*Homo Diluvii negator*,' to be coupled with Dr. Dowler's sub-cypress Indian, who dwelt on the site of New Orleans 57,600 years ago!"

In another paragraph he says:—

"These facts," (the discovery of human bones in a cave intermixed with the bones of extinct animals) "demonstrate that aboriginal man in America antedates the Mississippi alluvial, *because his bones are fossilized.*"

Again he asserts that—

"One human pelvis found near Natchez, by Dr. Dickeson, *is an undoubted fossil*,"

And again:—

"The drift abounds in fossil remains of animals; such as the elephant, mastodon, rhinoceros, hippopotamus, and other large mammalia. *These animals were destroyed by the same inundations which left the deposits we call drift;* yet the works and the remains of man have been found among them!"

In nearly every one of these statements so positively made, Dr. Usher is in direct opposition to other paleontologists. Sir Charles Lyell examined the "human pelvis" found by Dr. Dickeson, and concluded, from his examination, that the bones were *not* fossilized. It is still a matter of dispute with geologists whether human bones have ever been found in a fossil state. That they have been discovered in caves, mingled with the exuviæ of extinct races of animals, is what the mode of sepulture with many nations of men would have taught us to expect. Nor is it strange that human bones and implements should, now and then, be found buried under the drift, with the remains of the mastodon. It is easy to understand how, washed by the rains into ravines, or deposited on the spot in graves, they were brought to their final resting-place long after the mastodon had perished from the earth. Paleontologists have repeatedly examined the human remains found in such situations, and their decision has almost uniformly been, that the bones bore the marks of a comparatively recent origin.

But admit that human bones have been found fossilized; admit that the human race does date back further than biblical critics and geologists have hitherto conjectured, still the Mosaic account of man's origin upon earth remains unimpeached. We have only to change the chronology which has heretofore obtained, and allow all the time necessary to the modifications which his solid remains and the varieties of the race have undergone. The sacred historian is not responsible for the interpretation put upon his writings by critics and commentators. His chronology is a matter of inference. We have no idea that it was any part of his design to teach men those branches of science, which, by their own unassisted faculties, they are able to comprehend and master; but his mission was to impart to them those great truths, touching their origin and destiny, which their own reason could never have attained. As to the time that has elapsed since the creation of man, the christian, for all that we can see, may be indifferent how its duration is determined, whether at six thousand or three times six thousand years; the sublime central doctrine, of his creation, at the end of the work, and in the likeness of his Creator, is not affected by the decision.

But we must close this article, and in doing so we are ready unreservedly to express our opinion of "Types of Mankind." It is a work of learning and ability. It embraces a vast amount of information, and exhibits great labor and pains, both in its writers and publishers. But we are unable to see any great or desirable purpose its authors had in view in its publication. We can readily believe that it will help some, who wish to discredit revelation, to arguments for rejecting its teachings; but, we confess, we cannot divine any good that it is likely to accomplish.

